# Transcriptome profiling of induced susceptibility effects on soybean–soybean aphid (Hemiptera: Aphididae) interaction

**DOI:** 10.1186/s13104-019-4372-3

**Published:** 2019-06-10

**Authors:** Surendra Neupane, Adam J. Varenhorst, Madhav P. Nepal

**Affiliations:** 1 Department of Biology and Microbiology, South Dakota State University, Brookings, SD 57007 USA; 2 Department of Agronomy, Horticulture and Plant Science, South Dakota State University, Brookings, SD 57007 USA

**Keywords:** *Aphis glycines*, Transcriptome, RNA-seq, Soybean, Induced susceptibility

## Abstract

**Objectives:**

Soybean aphid (*Aphis glycines* Matsumura; SBA) is the most economically damaging insect of soybean (*Glycine max*) in the United States. One previous study demonstrated that avirulent (biotype 1) and virulent (biotype 2) biotypes could co-occur and interact on resistant (i.e., *Rag1*) and susceptible soybean resulting in induced susceptibility after 11 days of feeding. The main objective of this research was to employ RNA sequencing (RNA-seq) technique to compare the induced susceptibility effect of biotype 2 on susceptible and resistant soybean at day 1 and day 11 (i.e., both susceptible and resistant soybean were initially challenged by biotype 2 and the effect was monitored through biotype 1 populations).

**Data description:**

We investigated susceptible and *Rag1* transcriptome response to SBA feeding in soybean plants colonized by biotype 1 in the presence or absence of an inducer population (i.e., biotype 2). Ten RNA datasets are reported with 266,535,654 sequence reads (55.2 GB) obtained from pooled samples derived from the leaves collected at day 1 and day 11 post SBA infestation. A comprehensive understanding of these transcriptome data will enhance our understanding of interactions among soybean and two different biotypes of soybean aphids at the molecular level.

## Objective

Soybean aphid (*Aphis glycines* Matsumura; SBA) is the most economically damaging insect pest of soybean (*Glycine max*) in the United States (US) [1]. In the US, it is estimated that annual economic losses due to the SBA are approximately $4 billion [2]. Although host plant resistance to SBA exists, farmers rely on broad-spectrum foliar insecticide applications to reduce SBA populations [3]. The dependency on the use of chemical management has resulted in pyrethroid resistance in SBA populations in Iowa, Minnesota, North Dakota and South Dakota as well as the effects on non-target beneficial organisms [4, 5]. Host resistance to SBA is not widely adopted, which may partially be due to the presence of four SBA biotypes (i.e., biotype 1: avirulent, biotype 2: virulent to *Rag1*, biotype 3: virulent to *Rag2*, biotype 4: virulent to *Rag1*, *Rag2* and *Rag1 *+ *Rag2*) in the US [6–8]. Initial observations of SBA on resistant soybean were attributed to the presence of virulent biotypes [6–8]. However, Varenhorst et al. [6] demonstrated that inducer populations of avirulent (biotype 1) or virulent (biotype 2) biotypes improved conditions for subsequent (i.e., response) populations of biotype 1 or biotype 2 SBA on resistant (i.e., *Rag1*) and susceptible soybean, which is defined as induced susceptibility [9]. Furthermore, the induced susceptibility effect could be further categorized as feeding facilitation [10] (i.e., conspecific inducer improves host for conspecific response population) and obviation of resistance [11] (i.e., virulent inducer improves host susceptibility for avirulent response population). While induced susceptibility effects indicate that not all SBA observed on the resistant hosts are necessarily virulent [9], the mechanism of the induced susceptibility effects is yet to be characterized. Therefore, the major objective of this study was to use RNA sequencing (RNA-seq) to characterize induced susceptibility in soybean when a biotype 2 inducer is present.

## Data description

### Plant material and aphid biotypes

The data in this submission came from a greenhouse experiment using two genotypes of soybean (susceptible cultivar LD12-1583R, and resistant cultivar LD12-15813Ra with *Rag1* gene), and two SBA populations (biotype 1-avirulent and biotype 2-virulent [6]). A detailed overview of the experiment is provided in Supplementary file 1 and Figure S1 (Table 1).

**Table 1:** Overview of data files/data sets

Label	Name of data file/data set	File types (file extension)	Data repository and identifier (DOI or accession number)
Supplementary file 1	Methodology description	Word document (.dox)	10.6084/m9.figshare.7980176
Figure S1	A flow chart representing experimental methods	Image file (.tiff)	10.6084/m9.figshare.7980176
Figure S2	A flow chart showing the RNA-seq data analysis pipeline	Image file (.tiff)	10.6084/m9.figshare.7980176
Figure S3	The hierarchical clustering of top 3000 variable genes	Image file (.tiff)	10.6084/m9.figshare.7980176
Figure S4	The correlation between the samples using the top 75% genes	Image file (.tiff)	10.6084/m9.figshare.7980176
Figure S5	Quality metrics of *G. max* sequencing data. (a) Mean quality scores per position. (b) Per sequence quality scores. (c) GC content distribution. (d) Read length distribution	Image file (.tiff)	10.6084/m9.figshare.7980176
Data file 1	Control: No aphids; Susceptible soybean; Day 1; SRR8848027	fastq file (.fastq)	https://identifiers.org/ncbi/insdc.sra:SRR8848027
Data file 2	Control: No aphids; Susceptible soybean; Day 11; SRR8848028	fastq file (.fastq)	https://identifiers.org/ncbi/insdc.sra:SRR8848028
Data file 3	Control: No aphids; Resistant soybean; Day 1; SRR8848025	fastq file (.fastq)	https://identifiers.org/ncbi/insdc.sra:SRR8848025
Data file 4	Control: No aphids; Resistant soybean; Day 11; SRR8848026	fastq file (.fastq)	https://identifiers.org/ncbi/insdc.sra:SRR8848026
Data file 5	Inducer: None; Response: 15 biotype 1; Susceptible soybean; Day 11; SRR8848031	fastq file (.fastq)	https://identifiers.org/ncbi/insdc.sra:SRR8848031
Data file 6	Inducer: 50 biotype 2; Response:15 biotype 1; Susceptible soybean; Day 1;SRR8848032	fastq file (.fastq)	https://identifiers.org/ncbi/insdc.sra:SRR8848032
Data file 7	Inducer: 50 biotype 2; Response: 15 biotype1; Susceptible soybean; Day 11; SRR8848029	fastq file (.fastq)	https://identifiers.org/ncbi/insdc.sra:SRR8848029
Data file 8	Inducer: None; Response: 15 biotype 1; Resistant soybean; Day 11; SRR8848030	fastq file (.fastq)	https://identifiers.org/ncbi/insdc.sra:SRR8848030
Data file 9	Inducer: 50 biotype 2; Response: 15 biotype 1; Resistant soybean; Day 1; SRR8848023	fastq file (.fastq)	https://identifiers.org/ncbi/insdc.sra:SRR8848023
Data file 10	Inducer: 50 biotype 2; Response: 15 biotype 1; Resistant soybean; Day 11; SRR8848024	fastq file (.fastq)	https://identifiers.org/ncbi/insdc.sra:SRR8848024
Data file 11	Control: No aphids; Susceptible soybean; Day 1; GSM3717543	txt (.txt.gz)	http://identifiers.org/geo:GSM3717543
Data file 12	Control: No aphids; Susceptible soybean; Day 11; GSM3717544	txt (.txt.gz)	http://identifiers.org/geo:GSM3717544
Data file 13	Control: No aphids; Resistant soybean; Day 1; GSM3717545	txt (.txt.gz)	http://identifiers.org/geo:GSM3717545
Data file 14	Control: No aphids; Resistant soybean; Day 11; GSM3717546	txt (.txt.gz)	http://identifiers.org/geo:GSM3717546
Data file 15	Inducer: None; Response: 15 biotype 1; Susceptible soybean; Day 11; GSM3717547	txt (.txt.gz)	http://identifiers.org/geo:GSM3717547
Data file 16	Inducer: 50 biotype 2; Response: 15 biotype 1; Susceptible soybean; Day 1; GSM3717548	txt (.txt.gz)	http://identifiers.org/geo:GSM3717548
Data file 17	Inducer: 50 biotype 2; Response: 15 biotype1; Susceptible soybean; Day 11; GSM3717549	txt (.txt.gz)	http://identifiers.org/geo:GSM3717549
Data file 18	Inducer: None; Response: 15 biotype 1; Resistant soybean; Day 11; GSM3717550	txt (.txt.gz)	http://identifiers.org/geo:GSM3717550
Data file 19	Inducer: 50 biotype 2; Response: 15 biotype 1; Resistant soybean; Day 1; GSM3717551	txt (.txt.gz)	http://identifiers.org/geo:GSM3717551
Data file 20	Inducer: 50 biotype 2; Response: 15 biotype 1; Resistant soybean; Day 11; GSM3717552	txt (.txt.gz)	http://identifiers.org/geo:GSM3717552
Data file 21	The transformed transcript abundance counts for all the samples	Spreadsheet (.xlsx)	10.6084/m9.figshare.7980176
Data file 22	The hierarchical clustering of top 3000 variable genes	Spreadsheet (.xlsx)	10.6084/m9.figshare.7980176
Table S1	Statistics of the transcriptomic data using RNA-seq pipeline used in this study	Word document (.dox)	10.6084/m9.figshare.7980176

The supplementary materials (Supplementary file 1, Figure S1–S5, Data file 21, Data file 22, and Table S1) can be assessed openly on Figshare [19]. The raw RNA-seq data (.fastq files) are available for download on the SRA [20] and the raw transcript abundance counts (.txt.gz) are available on Gene Expression Omnibus (GEO) [21]

### RNA extraction, library preparation, and sequencing

Leaf samples collected at day 1 and day 11 from resistant and susceptible cultivars (non-infested, infested with inducer biotype 2: response biotype 1) were used to isolate RNA using PureLink RNA mini kit (Invitrogen, USA). Isolated RNA was treated with TURBO™ DNase (Invitrogen, USA) to remove any DNA contamination, following the manufacturer’s instructions. The RNA samples from three replicates were pooled in equimolar concentration, and RNA-seq libraries were sequenced on an Illumina NextSeq 500 at 75 cycles. Ten RNA libraries were prepared and sequenced with the sequencing depth ranging from 24,779,816 to 29,72,4913 reads (Data files 1–10; Table 1; Table S1).

### Quality control assessment

Quality control of reads was assessed using FastQC program (version 0.11.3) [12]. The FastQC results were visualized using MultiQC v1.3 [13]. Low quality bases (QC value < 20) and adapters were removed by trimming using the program Trimmomatic (version 0.36) [14]. The coding sequences (*Gmax: Gmax_275_Wm82.a2.v1.transcript_primaryTranscriptOnly.fa.gz*) were obtained from the Phytozome database and aligned using Salmon ver.0.9.1 [15] accessed from Bioconda [16] (Data files 11–20). A flow chart showing the RNA-seq data analysis pipeline is shown in Figure S2. The downstream analyses were conducted using iDEP 0.82 [17]. Read quants were filtered with 0.5 counts per million (CPM) in at least one sample. Quantified raw reads were transformed using regularized log (rlog), which is implemented in the DESeq 2 package [18] (Data file 21). The transformed data were subjected to exploratory data analysis such as hierarchical clustering (Figure S3; Data file 22) and the correlation between samples (Figure S4).

### Statistics of transcriptome data

The FastQC analysis showed Phred quality scores per base for all samples higher than 30, and GC content ranged from 45 to 46% with a normal distribution (Figure S5, Table S1). After trimming, over 99% of the reads were retained as the clean and good quality reads. Upon mapping these reads, we obtained high mapping rate ranging from 90.4 to 92.9%. Among the mapped reads, 85.8% to 91.9% reads were uniquely mapped. After filtering with 0.5 counts per million (CPM) in at least one sample and rlog transformation, a total of 37,468 genes (66.9% of original 55,983) were retained for transformation (Data file 21). The hierarchical clustering based on 3000 most variable genes, sample distances (Figure S3; Data file 22) indicated that sample clustering followed the time points of sample collection (i.e., Day 1 and Day 11). The correlation between the samples using the top 75% of genes showed in a range of 0.96–1 (Figure S4).

## Limitations

The quality filtering of downloadable raw fastq files is recommended before use. Kal’s z-test [22] integrated with CLC Genomics Workbench (https://www.qiagenbioinformatics.com/) and analysis guided by the reference genes could be used to study the differential gene expression for pooled samples with no replications.

## Data Availability

The raw fastq files were submitted to the National Center for Biotechnology Information and are available with accession numbers accession (SRR8848023–SRR8848032) under Bioproject PRJNA530958 (Project ID SRP190833) (Data files 1–10; SRR8848023, SRR8848024, SRR8848025, SRR8848026, SRR8848027, SRR8848028, SRR8848029, SRR8848030, SRR8848031, SRR8848032; SRP190833) [20]. The data could be retrieved using fastq-dump tool SRA toolkit (http://www.ncbi.nlm.nih.gov/sra). The file for raw transcript abundance counts for all the samples was deposited at the Gene Expression Omnibus (GEO) database, GSE129626 (Data files 11–20; GSM3717543, GSM3717544, GSM3717545, GSM3717546, GSM3717547, GSM3717548, GSM3717549, GSM3717550, GSM3717551, GSM3717552; GSE129626) [21]. The supplementary materials (Supplementary File 1, Figure S1–S5, Data file 21, Data file 22, and Table S1) can be assessed openly on Figshare (10.6084/m9.figshare.7980176.v5) [19]. Please see Table 1 and reference list for details and links to the data.

## Abbreviations

SBA: soybean aphids
RNA-seq: RNA sequencing
CPM: counts per million
*Rag*: resistance to *Aphis glycines*
